# The youngest are hit hardest: The influence of the COVID-19 pandemic on the hospitalization rate for children, adolescents, and young adults with anorexia nervosa in a large German representative sample

**DOI:** 10.1192/j.eurpsy.2022.2345

**Published:** 2022-11-21

**Authors:** Beate Herpertz-Dahlmann, Astrid Dempfle, Stefan Eckardt

**Affiliations:** 1 Department of Child and Adolescent Psychiatry, Psychosomatics and Psychotherapy, RWTH University, Aachen, Germany; 2 Institute of Medical Informatics and Statistics, Kiel University, Kiel, Germany; 3 Techniker Krankenkasse (Techniker Health Care Service), State Representation North Rhine-Westphalia, Düsseldorf, Germany

**Keywords:** Admission rates, adolescence, anorexia nervosa, childhood, COVID-19, pandemic

## Abstract

**Background:**

The COVID-19 pandemic has severely impacted the mental health of children and adolescents. Young people at risk for anorexia nervosa (AN) have been especially shown to be affected. There are no studies that have investigated the respective proportions of hospitalized children, adolescents, and young adults separately as well as of both sexes during the COVID-19 crisis.

**Methods:**

This study is based on the administrative data of the largest German statutory health insurance. All children (0–14 years) and adolescents (15–19 years) with a discharge diagnosis of typical and atypical AN according to the International Classification of Diseases (ICD)-10 were included. Admission rates per 10,000 person-years were calculated separately by sex and age group, based on admission numbers from the 9-month interval from January to September of 2019, 2020, and 2021 and the number of insured persons per sex and age group of each year.

**Results:**

The entire sample comprised approximately 4.7 million children and adolescents. There was a highly significant increase of 40% (relative risk (RR): 1.4; [1.27, 1.55]; *p* < 0.0001) in admission rates in the female children’s and the adolescents’ group (RR:1.32; [1.24, 1.41]; p< 0.0001) between the pre-COVID-19 and peri-COVID-19 periods in 2019 and 2021, respectively. Among males, hospitalization rates significantly increased in the children (RR: 1.69; [1.09, 2.62]; *p* < 0.02).

**Conclusions:**

Young people appear to be especially prone to develop AN during a crisis, such as with social isolation and school closures. Home-based or mobile pediatric services should be established to prevent this often chronic and disabling disorder in young patients.

## Introduction

Several studies have demonstrated that the COVID-19 pandemic has globally affected the mental health of children and adolescents but has had a particularly destructive potential effect on individuals either with or at risk for eating disorders (EDs) [1–3].

In a large-scale survey of 530 participants with different mental disorders, more than one-third of the adult patients with anorexia nervosa (AN) experienced a worsening of their symptoms [4]. In a systematic review of the impact of the pandemic on AN symptoms in various age groups, an increase in restrictive eating was especially prominent [5]. As has been demonstrated in other studies regarding the impact of the COVID-19 pandemic on global mental health, young people seem to be especially prone to the pandemic-associated psychological effects, because they lack “the mental capabilities of resilience and coping” [6]. In a small Austrian study, adolescents with AN reported reduced motivation to work on recovery, as well as increased exposure to triggering stimuli by media and social media and a disruption of treatment routines, which were all shown to interfere with the improvement of health [7]. Additionally, adolescents who were referred to a tertiary ED care program in Canada were significantly more medically unstable, required significantly more hospitalizations, and showed higher rates of self-reported impairment than patients who were referred 1 year previous [8].

Many of these studies refer to qualitative changes in ED symptom characteristics and severity. Other studies have reported on quantitative changes in health care provision for children and adolescents with an ED.

In a recent Israelian study, there was a 2.4-fold increase in admission rates of adolescent patients to the largest tertiary pediatric hospital in Israel between March 2020 and May 2021, in comparison to the pre-COVID-19 time period between 2015 and 2019. In this study, the mean age (14.63 years) and sex distribution were similar in both the pre- and peri-COVID-19 samples [9]. Accordingly, in Western Australia, a tertiary pediatric hospital observed a 104% increase in adolescents and children aged 15 years or less since the beginning of the COVID-19 pandemic [10]. A similar trend was also found in Eastern Australia after a primary decline in admissions in the spring of 2020 [11]. In a Canadian study involving 6 out of 10 pediatric centers with tertiary ED programs, monthly hospitalizations for newly diagnosed typical or atypical AN (according to the Diagnostic and Statistical Manual of Mental Disorders (DSM)-5) in individuals aged between 9 and 18 years nearly tripled from 7.1 cases to 20 cases/per month [12]. Moreover, a study from a specialized ED treatment unit in a large pediatric medical center in the United States reported a highly significant increase in admissions and readmissions during the COVID-19 pandemic [13]. All of these studies investigated incidence or admission rates that were limited to certain areas of the respective countries. Additionally, none of these studies differentiated between the two sexes and the proportions of the different age groups (children, adolescents, and young adults), although there is some evidence that the prevalence of AN in children had already increased in the pre-COVID-19 time period [14]. Thus, it was the aim of this study to explore the age- and sex-specific admission rates in a representative national cohort by using secondary data from the largest German health insurance company with more than one-third of German policyholders. The outcome of childhood AN is even worse than that of adolescent AN, with a high rate of school failure and lifetime psychiatric comorbidity; thus, early detection and intervention in this age group are of major importance [15, 16].

## Methods

This study was based on administrative data collected from a nationwide statutory health insurance association known as “Verband der Ersatzkassen” (VdEK), with which six health insurances throughout Germany are affiliated. In July 2021, the VdEK had more than 28 million members, with a market share of 38%. Thus, the VdEk is the largest statutory health insurance institution in Germany (https://www.vdek.com/presse/daten/b_versicherte.html, accessed January 16, 2022).

### Study design

The data were anonymously delivered to the main author for informal data sharing [17]. B.H.-D. and A.D. were allowed to use these data for scientific purposes.

#### Data

The dataset for this report was based on discharge data and diagnoses of inpatients who are members of the statutory health insurance institution “VdEK” from all psychiatric and general hospitals throughout Germany between January 2019 and September 2021. The entire dataset includes all insured persons in the age group of 0–19 years (born from 2000 until 2015) divided into two groups: (a) the childhood/preadolescent sample (ages: 0–14 years) and (b) the adolescent/young adult sample (ages: 15–19 years). These age groups were predefined by the data storage system of health insurance.

The time span between January 2019 and September 2021 was chosen to include the pre-COVID-19 time period and the two lockdowns that were caused by COVID-19. The first lockdown (including school closures) lasted from the end of March 2020 until May 2020 in Germany; the second lockdown started with minor restrictions in November 2020 followed by harsher restrictions including a closure of schools, which occurred from January 2021 until May 2021.

#### Patients

All of the patients who were diagnosed with AN according to the ICD-10 (F 50.0) and corresponding to the German guidelines for EDs (https://www.awmf.org/leitlinien/detail/ll/051-026.html, accessed on February 2, 2022), involving a body mass index (BMI) at or below the 10th age-adapted percentile until the age of 17 years, and a BMI below 17.5 kg/m^2^ in the 18- to 19-year-old group, and those with atypical AN (ICD-10: F 50.1; AN without amenorrhea or with another missing criterion) were assigned to the study group. In most cases, diagnoses were made by child and adolescent psychiatrists and/or pediatricians in the childhood group, as well as by psychiatrists, child and adolescent psychiatrists, specialists in psychosomatic medicine, and specialists in internal medicine in the adolescent and young adult groups. All professionals were clinicians working in a hospital.

### Data analysis

Admission rates per 10,000 person-years were calculated separately by sex and age group, based on hospital admission numbers from the 9-month interval of January to September of each year (as available for all 3 years) and the number of insured persons (per sex and age group) of each year. Cases were only counted once, for example, a transfer from a general hospital to a psychiatric or psychosomatic hospital and vice versa was not taken into account for the statistical analysis. According to recent German census data, 51.32% of the population in the age group of 0–19 years was male. The 95% confidence intervals for admission rates were based on Wilson’s approximation [18, 19]. Admission risk ratios for comparing risks across years are also based on the January to September data and are presented with 95% confidence intervals and *p*-values from Chi-squared tests. Data analysis was performed by using the package epiR in R [20].

## Results

The entire sample comprised approximately 4.7 million children and adolescents (2019: *n* = 4,660,566; 2020: *n* = 4,652,625; 2021: *n* = 4,645,135) (Table 1). There were only small differences in the number of insured patients between 2019, 2020, and 2021, with a slightly increasing number between 2019 and 2021 in the childhood group and a slightly decreasing number in the adolescent/young adult group. The youngest patients with a diagnosis of AN were 6 years old. Although there was a significant decrease in hospital admissions for female patients with a diagnosis of typical or atypical AN in the childhood group between the time periods of January 2020 until September 2020 versus the same time span in 2019 (RR: 0.83 [0.74, 0.93]; *p* = 0.001) (Table 1), there was a highly significant increase of 40% in the female children group during the months of January until September 2021 in comparison to January until September 2019 (specifically, between the COVID-19 time period and the pre-COVID-19 time period) (RR: 1.40 [1.27, 1.55]; *p* < 0.0001; Table 1). In the male children’s group, there was a significant increase between the pre-COVID-19 period and the COVID-19 period from January to September 2020 (RR: 1.67 [1.08, 2.59]; *p* = 0.026), as well as between the same time period of months between 2019 and 2021 (RR: 1.69 [1.09, 2.62] *p* = 0.02). However, the admission rates for males were very low (Table 1).

**Table 1.** Admission rates for children (0–14 years) with AN.

		2019	2020	2021	∆ 2020 versus 2019 (*n*) Admission risk ratio	∆ 2021 versus 2020 (*n*) Admission risk ratio	∆ 2021 versus 2019 (*n*) Admission risk ratio
Insured persons Males and females (*n*)		3.409.969	3.438.287	3.463.211			
Females							
Cases	January–December	887	843	–	−44	–	–
	January–September	670	560	953	−110	393	283
Admission rate		5.38	4.46	7.54	0.83	1.69	1.40
		(4.99, 5.80)	(4.11, 4.85)	(7.07, 8.03)	(0.74, 0.93)	(1.52, 1.88)	(1.27, 1.55)
					*p* = 0.001	*p* < 0.0001	*p* < 0.0001
Males							
Cases	January–December	47	73	–	26	–	–
	January–September	32	54	55	22	1	23
Admission rate		0.24	0.41	0.41	1.67	1.01	1.69
		(0.17, 0.34)	(0.31, 0.53)	(0.32, 0.54)	(1.08, 2.59)	(0.69, 1.47)	(1.09, 2.62)
					*p* = 0.026	*p* = 1	*p* = 0.02

*Note*: The age groups were predefined by the data storage system of the health insurance institution. The youngest children in the female group were two 6-year-old girls in 2019 and 2020 diagnosed with atypical AN, respectively; in 2021, the youngest girls with typical and atypical AN were 9 years of age. In the male group, the youngest boy was 9 years in each year.

When subdividing our sample into typical and atypical AN, the percentage of atypical AN in the childhood group was as follows: 26.2% in 2019, 19.2% in 2020, and 19.1% in 2021.

Admission rates of children increased in psychiatric and general hospitals; interestingly, in comparison to psychiatric hospitals, the admission rates in general hospitals increased even more in the peri-COVID-19 period than in the pre-COVID-19 time (Table 2).

**Table 2.** Readmission rates and rates of psychiatric versus general hospital admissions for children (0–14 years) with AN.

	2019	2020	2021	∆ 2020 vs 2019 (*n*) (Re-)admission risk ratio	∆ 2021 vs 2020 (*n*) (Re-)admission risk ratio	∆ 2021 vs 2019 (*n*) (Re-)admission risk ratio
Females and males together						
Readmissions (Jan. – Sept.)	23	25	41			
Readmission rate	3.39	4.24	4.24	1.25	1.00	1.25
	(2.27, 5.03)	(2.89, 6.19)	(3.14, 5.70)	(0.72, 2.18)	(0.61, 1.63)	(0.76, 2.07)
				*p* = 0.52	*p* = 1	*p* = 0.45
Admissions to a psychiatric hospital (Jan. – Sept.)	423	352	508			
Psychiatric admission rate	1.65	1.37	1.96	0.83	1.43	1.18
	(1.50, 1.82)	(1.23, 1.52)	(1.79, 2.13)	(0.72, 0.95)	(1.25, 1.64)	(1.04, 1.35)
				*p* = 0.009	*p* < 0.0001	*p* = 0.01
Admissions to a general hospital (Jan. – Sept.)	280	262	500			
General hospital admission rate	1.09	1.02	1.92	0.93	1.89	1.76
	(0.97, 1.23)	(0.90, 1.15)	(1.76, 2.10)	(0.78, 1.10)	(1.63, 2.20)	(1.52, 2.04)
				*p* = 0.41	*p* < 0.0001	*p* < 0.0001

In 2019 there is one case more in the sum of admissions to hospital (first column table 1b) than in the global sum of males and females (first column Table 1a). In one case information on sex was missing.

There was no significant difference in readmission rates during the observation time.

In the adolescent and young adult female group, there was a decrease in hospitalizations amounting to 12% between January and September 2020, compared with the same time period of months in 2019 (RR: 0.88 [0.82, 0.95]; *p* = 0.0009) (Table 3); in contrast, there was a highly significant increase in the period between January and September 2021 in comparison to the same period 2020 (RR: 1.50 [1.40, 1.61]; *p* < 0.0001), as well as a less considerable (but also highly significant) increase of 32% of admission rates between 2019 and 2021 (RR: 1.32 [1.24, 1.41]; *p* < 0.0001) (Table 3). In the male adolescent group, admission rates were very small and nearly stable between 2019 and 2021 (Table 3).

**Table 3.** Admission rates for adolescents (15–19 years) with AN.

		2019	2020	2021	∆ 2020 versus 2019 (*n*) Admission risk ratio	∆ 2021 versus 2020 (*n*) Admission risk ratio	∆ 2021 versus 2019 (*n*) Admission risk ratio
Insured persons Males and females (*n*)		1.250.597	1.214.338	1.181.924			
Females							
Cases	January–December	2.013	1.875	–	−138	–	–
	January–September	1.551	1.329	1.937	−222	608	386
Admission rate		33.97	29.98	44.89	0.88	1.50	1.32
		(32.32, 35.70)	(28.41, 31.63)	(42.94, 46.93)	(0.82, 0.95)	(1.40, 1.61)	(1.24, 1.41)
					*p* = 0.0009	*p* < 0.0001	*p* < 0.0001
Males							
Cases	January–December	90	81	–	−9	–	–
	January–September	74	58	69	−16	11	−5
Admission rate		1.54	1.24	1.52	0.81	1.22	0.99
		(1.22, 1.93)	(0.96, 1.60)	(1.20, 1.92)	(0.57, 1.14)	(0.86, 1.73)	(0.71, 1.37)
					*p* = 0.26	*p* = 0.30	*p* = 1

The percentage of atypical AN in the adolescent group was 31.2% (2019), 31.2% (2020), and 34.5% (2021), respectively.

Similar to the children’s group the admission rates in general and psychiatric hospitals both increased between the pre-COVID-19 and COVID-19 period, however, even more in general hospitals (Table 4). According to our findings in the younger group, readmission rates did not differ in the respective time period. However, readmission rates in this dataset only referred to patients of the same year; thus, we cannot draw conclusions about patients who were readmitted in different years.

**Table 4.** Readmission rates and rates of psychiatric versus general hospital admissions for adolescents (15–19 years) with AN.

	2019	2020	2021	∆ 2020 versus 2019 (*n*) (Re-)admission risk ratio	∆ 2021 versus 2020 (*n*) (Re-)admission risk ratio	∆ 2021 versus 2019 (*n*) (Re-)admission risk ratio
Females and males together						
Readmissions (January–September)	69	66	70			
Readmission rate	4.43	5.00	3.62	1.13	0.72	0.82
	(3.52, 5.57)	(3.95, 6.31)	(2.87, 4.54)	(0.81, 1.57)	(0.52, 1.01)	(0.59, 1.13)
				*p* = 0.53	*p* = 0.06	*p* = 0.25
Admissions to a psychiatric hospital (January–September)	1138	892	1247			
Psychiatric admission rate	12.13	9.79	14.07	0.81	1.44	1.16
	(11.45, 12.86)	(9.17, 10.46)	(13.31,	(0.74, 0.88)	(1.32, 1.57)	(1.07, 1.26)
			14.87)	*p* < 0.0001	*p* < 0.0001	*p* = 0.0003
Admissions to a general hospital (January–September)	488	495	759			
General hospital admission rate	5.20	5.44	8.56	1.04	1.58	1.65
	(4.76, 5.69)	(4.98, 5.94)	(7.97, 9.19)	(0.92, 1.18)	(1.41, 1.76)	(1.47, 1.84)
				*p* = 0.52	*p* < 0.0001	*p* < 0.0001

*Note*: Admission rates (overall and separately for admissions to psychiatric and general hospitals) per 10,000 person-years, readmission rates per 100 patients with AN (females and males together), all based on January–September data (as available for all 3 years), together with 95% confidence interval. (Re-)Admission risk ratios also based on January–September data, together with 95% confidence interval and *p*-value from χ^2^-test. According to recent German census data, 51.32% of the population in the age group ≤19 years are male.

Overall, the increase in admission rates between 2019 and 2021 in the younger group was comparable (if not slightly higher) than that in the adolescent group (Tables 1 and 3).

**Figure 1.**
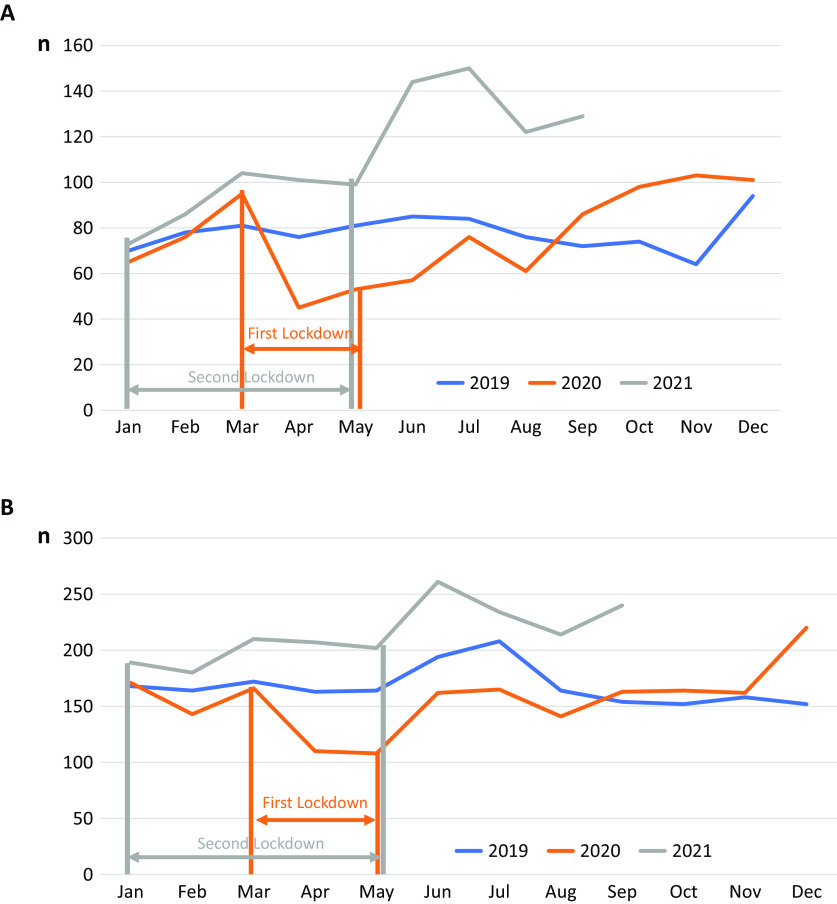
(A) Number of hospitalizations/month in the childhood group (0–14 years) during the period 2019–2021. Number of hospitalizations/months for patients of both sexes with typical and atypical anorexia nervosa (ICD-10: F 50.0, F 50.1) during the years 2019, 2020, and 2021. Data are based on the statutory health insurance set of a large health insurance in Germany including all insured persons in the age group of 0–19 years. (B) Number of hospitalizations/month in the adolescent/young adult group (15–19 years) during the period 2019–2021.The two periods of lockdowns are indicated by bars. The time of the first lockdown in 2020 is marked in orange and the time of the second lockdown in 2021 is marked in gray.

In relation to the monthly admission rates during the two lockdowns, there was a decrease in both age groups during the first lockdown and a distinct increase after 4–5 months (Figure 1A,B). High admission rates were also observed during the second lockdown from November 2020 to May 2021, with another significant increase in hospitalizations occurring after the end of the restrictions in both age groups.

## Discussion

Our results confirm the results of other recent investigations that observed a COVID-19-induced increase in hospitalizations because of AN in young people (e.g., [9, 12]). However, in our opinion, this is the first study to demonstrate that this increase was at least as high in the children’s and young adolescent groups, compared to the older adolescent and young adult groups. Moreover, we were the first to demonstrate the effect of COVID-19 on hospitalization rates in a nationwide representative sample. In addition, we could also demonstrate a greater growth in admission rates to general hospitals in comparison to psychiatric hospitals. The percentage of atypical AN corresponded to that of other investigations in the pre-COVID-19 era [21] and did not change during the observation period.

The comparable increase in admission rates in both age groups contrasts with the expectation based on the pre-COVID-19 time period, when admission rates in the children and preadolescent age groups were much lower [14, 22]. This emphasizes the detrimental effect of the COVID-19 pandemic on the youngest age groups.

The majority of previous studies did not evaluate the impact of COVID-19 on different pediatric age groups. Corresponding to our own results in the childhood group, Haripersad et al. [10] investigated patients younger than 16 years and found a significant increase during the COVID-19 pandemic, compared to the previous 3 years.

The increase in admissions in both age groups cannot be explained in our study by a higher number of readmissions during the COVID-19 era, as postulated by [13]. In our investigation, readmission rates did not differ significantly between three consecutive years; thus, a COVID-19 induced new onset of the ED or a more severe course of the disorder followed by hospitalization is more probable.

As in non-COVID-19 time periods, there were still only a few boys suffering from AN; however, there was a significant increase in the male childhood/preadolescent group.

There is likely more than one explanation for why the COVID-19-associated increase was surprisingly high in the youngest age group. Before the COVID-19 pandemic, an increase in childhood AN was reported by several epidemiological surveys. In Norway, a population-based health care register study revealed a significant annual increase in typical and atypical AN in 10- to 14-year-old girls between 2010 and 2016, whereas the rate of adolescent AN remained mostly stable during this 7-year time period [14]. An increase in incidence was also reported in an epidemiological survey from the United Kingdom and Ireland [23]. In a Danish psychiatric registry study, the average age period at the onset of illness declined from 16 to 19 years of age to 12 to 15 years [24]. A very recent literature review on the incidence and prevalence of AN confirmed an increase in the disorder in children below 15 years of age [25]. Correspondingly, an increasing trend in admission rates was observed for patients below the age of 15 years in England [22] and Germany [26].

However, all of these epidemiological data originated in the time period before the COVID-19 pandemic and do not explain a 40% increase in the children and young adolescent groups between 2019 and 2021. A more appropriate justification could involve a compensatory increase in hospital admissions after the COVID-19-induced “dip” in hospitalizations at the beginning of 2020, which was also found in other studies. At that time, many parents were afraid to bring their children to the hospital because of fear of contagion [27]. However, the increase in admission rates between January and September 2020 and during the same time span in 2021 is much higher than the decline in admissions between 2019 and 2020. Moreover, the increase between the pre-COVID-19 era in 2019 and the peri-COVID-19 era in 2021 is too high to be solely explained by a compensatory increase. Additionally, there was a small but significant increase in the admission rates for boys at or below 14 years of age with AN. Previous studies have shown that the difference in prevalence between female and male AN is smaller in childhood than in adolescence [28, 29]; therefore, younger boys may be more vulnerable to environmental burdens.

A more plausible explanation for the considerable increase in admission rates is the impact of the COVID-19 pandemic itself [30]. Our data demonstrated the highest increase in admissions at 1–2 months after the end of the strict restrictions. This is very similar to other large epidemiological studies assessing COVID-19-associated mental health symptoms in children and adolescents. In a United Kingdom-based longitudinal online survey of mental health in children, adolescents, and their parents, the highest incidences of symptoms were described when high levels of restrictions were enforced and schools were closed (Co-SPACE study, [31]). Most likely, weight loss started during the most severe restriction period and could not be stopped thereafter.

Although admission rates increased both in psychiatric and general hospitals, the increase in general hospitals was relatively higher. One plausible explanation could be that—because of the rise in admissions—child and adolescent psychiatric beds were occupied, and patients had to use general hospital care.

In addition, especially parents of younger children might have preferred general hospital treatment in comparison to psychiatric treatment, which could have contributed to the high general hospital admission rates in the younger group.

According to previous studies [7], young patients with AN reported that they had lost their daily life structure, especially during school closures [32]; according to Zeiler et al. [7], they complained of participating in fewer outdoor activities, including sports, during the lockdowns, which then triggered weight phobia. In addition, they had more spare time and experienced more boredom, which led to increased activity in social media, especially on websites glorifying slimness and a well-trained body [33]. During the COVID-19 time period, a higher activity on social media was observed in both adolescents and children (International Central Institute for Youth and Educational Television [34]).

Several studies point to a lockdown-induced weight gain in children and adolescents [35]. In a previous investigation, we reported that patients had assumed to have put on weight during the first lockdown and felt bullied because of “fatness” after their return to school, thus leading to increased efforts to lose weight [32]. Accordingly, it is well known that intended weight loss may lead to reduced eating and exaggerated participation in sports with the consequence of starvation.

Only a few studies have separately assessed the development of mental disorder symptoms during the COVID-19 pandemic in children and adolescents. Of those, the majority of the studies observed a higher increase in the younger group compared to the older group. In the longitudinal Co-SPACE study, parent-rated symptoms were higher in the preadolescent group than in the adolescent group. Parents assumed that the isolation of children was even more pronounced than in adolescents [31]. In a recent study by the same group, a 10% increase in the number of preadolescents meeting possible caseness criteria for emotional symptoms between the beginning of the COVID-19 pandemic and 3 months later was observed [36], whereas a small reduction in symptoms translating to 3% was observed in adolescents.

Although we do not know the mean age of the younger patients group of our sample, it is highly probable that rather the older children and preadolescents (and not the younger ones) suffered from AN [37] and were admitted to the hospital. In a large German epidemiological study assessing the effect of the pandemic on mental health, the percentage of children reporting low health-related quality of life was significantly higher in 11- to 13-year-olds than in 14- to 17-year-olds [1]. As has been shown by previous studies, the onset of puberty typically occurring during this age span is associated with an increase in mental health problems, especially in girls [38]. Thus, the effect of the COVID-19 crisis on mental health in this age group may have been especially detrimental.

## Limitations and Strengths

There were some major limitations to our study. First, our results only refer to statutory health insurance data which could not be controlled for disorder-specific classification criteria. However, with the help of these insurance data, we could investigate a very large sample size. Second, details of the admission data of our sample, such as the mean age, mean current BMI, duration of illness, medical instability, emergency room admission, eating disordered behavior, and numbers of previous inpatient or outpatient treatments (except for readmission in the same year), were not available. However, as we did not see any significant difference in readmission data for three consecutive years, it is highly improbable that there was an increase in readmission rates. Third, because of predefined age groups in the data storage system of the health insurance institution, we could not exclusively assess admission rates in the vulnerable children’s group between 6 years and 14 years. Fourth, because of missing clinical data, we cannot exclude that children were more likely to be admitted to the hospital than adolescents or young adults [39]. However, in our opinion, this is the only study that has differentiated between admission rates of children/preadolescents and adolescents/young adults and assessed the nationwide admission rates of this age group independent of region, supply structure of the hospital (primary, secondary, or tertiary care), and medical discipline.

## Conclusions

Hospital admissions due to juvenile AN have dramatically increased during the COVID-19 pandemic. Specifically, there has been an alarming increase in admissions of childhood AN. The causes are not quite clear. Most likely, the loss of everyday life structure associated with school closures produced a feeling of loss of control in vulnerable individuals, thus resulting in counteractive measures, such as restrictive eating and excessive exercising. Girls in late childhood and at the onset of puberty seem to be particularly at risk. Early onset AN is associated with a poor outcome. Moreover, COVID-19-induced suspension of routine pediatric care, which had been established in Germany many years ago, has likely resulted in less control of growth parameters such as weight and height, thus contributing to the onset and deterioration of a restrictive ED with the consequence of hospital treatment. We urgently need care delivery systems that reach children and adolescents with mental disorders at home and at school, such as mobile health units [40], to prevent and diagnose debilitating disorders such as childhood AN at earlier times.

## Data Availability

Restrictions apply to the availability of these data, which were used under license for this study.
